# Identification and analysis of differentially expressed (DE) circRNA in epididymis of yak and cattleyak

**DOI:** 10.3389/fvets.2023.1040419

**Published:** 2023-02-07

**Authors:** Chunhai Li, Yan Yan, Cheng Pan, Michael Adjei, Khuram Shahzad, Peng Wang, Meilan Pan, Kerui Li, Ye Wang, Wangsheng Zhao

**Affiliations:** ^1^School of Life Science and Engineering, Southwest University of Science and Technology, Mianyang, Sichuan, China; ^2^College of Life Sciences, Yan'an University, Yan'An, Shaanxi, China; ^3^Department of Biosciences, COMSATS University Islamabad, Islamabad, Pakistan; ^4^Sichuan Key Laboratory of Conservation Biology on Endangered Wildlife, Chengdu Research Base of Giant Panda Breeding, Chengdu, Sichuan, China

**Keywords:** cattle, cattleyak, DE circRNA, epididymis, transcriptome sequencing

## Abstract

Circular RNAs (circRNAs), as endogenous non-coding RNA with unique closed ring structure, is closely related to animal reproduction, and understanding the expression of circRNA in yak and cattleyak epididymal tissues is of great significance for understanding cattleyak sterility. Based on this, we screened and identified the differentially expressed circRNA in the epididymis of three yaks and two cattleyak. A total of 1,298 circRNAs were identified in the epididymis of yak and cattleyak, of which 137 differentially expressed (DE) circRNAs and the functions of some of them were elucidated in this research, as well as qPCR verification to 6 circRNAs from the 137 DE circRNAs. Gene Ontology (GO) enrichment analysis suggested that DE circRNAs were mainly related to metabolic process, development process, immune system process, reproductive process, reproduction, biological adhesion and growth. COG classification analysis showed that the DE circRNAs derived genes were mainly related to replication, recombination and repair. KEGG pathway analysis suggested that DE circRNAs were mainly involved in RNA degradation. In addition, we also screened Bta-mir-103, which is a circRNA binding miRNA related to sperm activity.

## 1. Introduction

A new group of circular RNAs (circRNAs) with covalently closed-loop structures have gained attention recently as novel molecules of gene regulation as other non-coding RNAs. CircRNAs were more stable than linear RNAs under RNAse R treatment because of the lack of free 5' and 3' ends (1). According to their sequence construction and sources from the genome, the so far discovered circRNAs can be mainly classified into three categories, such as exonic circRNAs, intronic circRNAs (ciRNAs) and exon-intron circRNAs (EIciRNAs). Exonic circRNAs are mainly found in the cytoplasm to act as miRNA sponges (2, 3). CiRNAs and EIciRNAs are speculated as efficient regulators in *cis/trans* for their parental genes in the nucleus (4, 5). Some circRNAs may also regulate gene expression by competitive alternative RNA splicing (6), or by acting as translation templates (7). Their main role is to modulate microRNAs (miRNAs) and protein availability working as sponges (8). This tethering activity inhibits miRNAs, protecting their mRNA targets from degradation. For this reason, circRNAs are part of the competitive endogenous RNA (ceRNA) network (ceRNET). Besides tethering activity, circRNAs also regulate transcription of their host genes (5) and splicing of their cognate mRNAs (9).

Cattleyak is a hybrid breed of male yak and female cattle. It shows obvious heterosis in meat and dairy quality and is also the main economic source of herdsmen in Plateau areas of China. However, the male offspring cattleyak is born through spermatogenesis dysfunction (10), which leads to their infertility. Our research team has studied the expression of miRNA (11) in yak epididymis and the expression of miRNA (12) and lncRNA (13) in cattleyak epididymis, and identified the proteins related to sperm maturation in yak and cattleyak epididymis by comparing iTRAQ (14) proteomics. Many scientists have carried out cellular and molecular level research to determine various factors causing the infertility. The cellular level research (15) shows that cattleyak infertility may be caused by meiotic chromosome pairing anomalies, which results in the stagnation of spermatogenesis in spermatogonial stem cells (SSCS) or primary spermatocyte phase. On the other hand, the molecular level (16), research shows that DNA hypermethylation and low expression of spermatogenesis related genes are the cause of male cattleyak infertility. During spermatogenesis, spermatozoa formed in the testis are carried to the epididymis, where post-testicular maturation of the sperm cells take place. Post-testicular maturation of spermatozoa mainly involves motility acquisition and the competence to undergo capacitation, which leads to fertilization of an egg (17, 18). The epididymis is a complex convoluted tube that connects efferent ducts to the vas deferens in the male reproductive tract (19, 20). The function of the epididymis is crucial to the maturation of normal sperm, as it is through this organ that sperms gain full activity for reproduction (21).

In the past couple of years, few studies have been conducted on circRNAs and have identified their critical role in sperm development (22, 23). CircRNAs have been identified in the testis of human (22) and cattle (23). However, none of such studies have been demonstrated in the epididymis. Therefore, epididymal based study may elucidate the causes of infertility in cattleyak and also enhance our understanding of molecular mechanisms underlying in this region. As it is mentioned earlier, circRNAs analysis on epididymal based study is yet to be explored. Therefore, we used RNA-seq to study the important role of circRNA in the regulation of gene expression in the epididymis of cattleyak and yak, so as to lay a foundation for yak and cattleyak breeding. In addition, circRNA: X:132347491|132372215 and 20:2680598|2714639 associated with spermatogenesis were successfully screened (24–27).

## 2. Materials and methods

### 2.1. Sample collection and treatment

Three adult male yak and three cattleyak testicles with 36 months of age, physique and health status were collected in the abattoir of Hongyuan County, Aba Prefecture, Sichuan Province. The epididymis was cleaned three times with Hanks solution and was separated from testis.

### 2.2. Total RNA isolation

Extraction of Total RNA from yak and cattleyak epididymis tissue was performed according to the instructions of Trizol reagent. The total RNA from yak and catleyak epididymis (caput, corpus and cauda) were extracted. The integrity of RNA was detected by 0.8 % agarose gel electrophoresis apparatus and the quality of RNA was detected by SpectraMax Quick Drop ultrafine spectrophotometer and Qubit 4.0. The extracted RNA was stored in an ultra-low temperature refrigerator (−80°C).

### 2.3. RNA sample detection

Nanodrop, Qubit 2.0, Agilent 2100 and electrophoresis were used to detect the purity, concentration and integrity of RNA samples. These instruments were used to determine whether there was genomic DNA contamination, so as to ensure the use of qualified samples for circRNAs sequencing.

### 2.4. Construction of RNA library

After qualified samples were detected, library construction was carried out and the main processes were as follows: rRNAs of samples were removed using Epicenter Ribo-ZerotM kit; fragmentation buffer was added to randomly break the rRNA- depleted RNA. The depleted RNA was cloned into the first cDNA strand using random hexamers. The second cDNA strand was synthesized by adding buffer, dATP, dUTP, dCTP, dGTP, RNAse H and DNA polymerase I and the cDNAs were purified using AMPure XP Beads. The purified double-stranded cDNA was repaired, A was added and sequenced. Then AMPure XP Beads were used for fragment size selection. Finally, the U chain was degraded and the cDNA library was enriched by PCR.

A qualified sequencing library is a necessary condition for sequencing. To ensure the quality of the library, the quality of the sequencing library is evaluated using the following two different perspectives: (1) The randomness of CircRNA fragmentation and the degradation of CircRNA is evaluated by checking the distribution of inserted fragments in genes; (2) The dispersion degree of the inserted fragment length is evaluated by the length distribution of the inserted fragment.

### 2.5. Library quality control

After library construction, the quality of the library was tested and machine sequencing was performed only after the test results meet the requirements. The detection methods proceeded: (1) Qubit2.0 was used for preliminary quantification; Agilent 2100 was used to detect the insert size of the library and the next experiment was carried out only after the insert size met expectations. (2) The effective concentration of the library was accurately quantified by q-PCR (effective concentration of library >2 nM) to complete library inspection.

The original sequence obtained by sequencing contains joint sequence or low-quality sequence. In order to ensure the accuracy of information analysis, it is necessary to conduct quality control on the original data to obtain high-quality sequences (i.e., Clean Reads). The quality control standard of the original sequence is: (1) Removed the read with connector; (2) Filtered and removed low quality data to ensure data quality; (3) Removed the reads containing N (unable to determine the base information) whose proportion is >5%. The high-quality read obtained after the above series of quality control are called Clean Data, which is also provided in FASTQ format.

### 2.6. Bioinformatics analysis

Raw data were filtered to remove joint sequences and low-quality reads to obtain high-quality clean data. The clean data was aligned against the specified reference genome to obtain the mapped data. Based on the mapped data, the quality assessment of sequencing library such as insert length test and randomness test were carried out. CIRI software (28) prediction circRNA, circRNA binding site analysis and CircRNA expression level analysis of different samples. RNAhybrid was used to predict the target gene of circRNA. The circRNA source genes (CSGs) were annotated with relevant functions, and the CSGs were analyzed with standardized biological annotation through GO database to compare the enrichment trend of circRNA source genes and all other genes. The CSGs were classified into lineal homologies using COG database. The function of CSGs was further interpreted by analyzing the KEGG pathway annotation of CSGs.

### 2.7. qPCR validation of DE circRNAs

Based on the verified miRNA expression in our team's already published papers (29, 30), among the 137 DE circRNAs obtained in this study, six of them (see Table 1) were screened to test the differential expression of circRNA by qPCR, and the relationship between circRNA, miRNA and mRNA was verified. cDNA is obtained by reverse transcription from total RNA using SuperScript™ III reverse transcriptase (Invitrogen, Carlsbad, CA, USA). The SYBR Green assay was used in the CFX Connect™ real-time PCR detection system (Bio-Rad, Munich, Germany), and SYBR Premix Ex Taq (TaKaRa, Dalian, China) was used for qPCR validation using *GAPDH* as the internal reference gene. The qPCR reaction system was 95°C for 5 min, followed by 40 cycles of 95°C for 10 s, 60°C for 10 s and 72°C for 20 s. Each circRNA has two biological replicates. Data were processed using the 2^−Δ*ΔCt*^ method and statistical analysis was performed using Student's *t-*test in Simplot 11.2 software to compare the differences between yak and cattleyak epididymal samples at *p*<*0.05*. Primers for qPCR are listed in Table 2.

**Table 1:** Six DE circRNA information.

**CircRNA ID**	**miRNA**	**mRNA**	**Gene**	**FDR**	**Regulated**
1:69644514|69650504	unconservative_12_372712	ENSBTAG00000002640	*KALRN*	0.1928	Up
21:21565870|21570999	unconservative_14_491632	ENSBTAG00000006141	*-*	0.0728	Up
3:78748144|78779079	unconservative_11_310424	ENSBTAG00000005442	*DNAI4*	0.0689	Up
19:24088810|24112268	unconservative_13_402958	ENSBTAG00000016806	*PAFAH1B1*	0.4301	Down
5:48639209|48702833	unconservative_22_1152540	ENSBTAG00000044017	*MSRB3*	0.4301	Down
6:117810462|117832269	bta-miR-504	ENSBTAG00000019584	*LOC532207*	0.0915	Down

**Table 2:** Primers for qPCR validation of RNA-seq analysis results.

**CircRNA ID**	**CircRNA primer (5'-3')**	**CircRNA primer (3'-5')**
1:69644514|69650504	GCAGGTGTTAGATTGGATTG	CCGCATTGGTGTATGTATTC
21:21565870|21570999	ATCAACAAGATGGTGAACTC	CTAAGTGGATGGTAGATGGT
3:78748144|78779079	TTGGCTGTTGGCTATGGA	GACTCTGATAAGTACGCTCTG
19:24088810|24112268	ATTGAGTGGTCATAGGAGTC	CAGAACATGAAGCCAGAAG
5:48639209|48702833	AATACCATGTCACTCAGGA'	AACAACGGAGTTCCACAA
6:117810462|117832269	GTTCTTCAACGCCTCACT	GCATGTCCGACGAATAGG

## 3. Results

### 3.1. Quality control of data

In this study, we have used six epididymal tissue samples of control (yak) and treatment (cattleyak) to construct cDNA libraries. Of the five tissue samples; three from yak (named L1, L2, and L3) and two from cattleyak (named L4 and L5) were completed, and then subjected to RNA-sequencing analysis. We obtained 99.21 Gb clean data from all five epididymal tissue samples. The clean data of each tissue sample reached 17.01Gb and the Q30 base percentage was not < 92.43% (Table 3). The efficiency of aligning reads with specified reference genome UMD3.1 of each tissue sample ranged from 99.98 to 99.99% (Table 4). The data utilization rate is normal and the selected reference genome can meet the needs of subsequent analysis. All data had good biological replicates and met the analytical requirements (Table 5).

**Table 3:** Evaluation statistics of sample sequencing data.

**Samples**	**Reads**	**Base number**	**GC content**	**%≥Q30**
L01	65,211,045	19,525,872,986	46.06%	92.43%
L02	72,966,371	21,854,490,746	45.39%	92.82%
L03	56,907,177	17,008,466,848	44.66%	92.86%
L02	63,458,540	18,998,263,402	43.87%	93.85%
L03	72,916,799	21,822,906,020	44.82%	93.70%

**Table 4:** Statistics of comparison results between clean data and reference genome.

**BMK-ID**	**Total reads**	**Mapped reads**	**Unique map reads**	**Multiple map reads**	**Reads map to '+'**	**Reads map to '–'**
L01	130,422,090	130,406,446 (99.99%)	123,959,297 (95.04%)	6,447,149 (4.94%)	63,938,139 (49.02%)	63,969,757 (49.05%)
L02	145,932,742	145,915,282 (99.99%)	139,456,130 (95.56%)	6,459,152 (4.43%)	71,605,926 (49.07%)	71,645,986 (49.10%)
L03	113,814,354	113,791,160 (99.98%)	107,737,248 (94.66%)	6,053,912 (5.32%)	55,844,163 (49.07%)	55,878,230 (49.10%)
L04	126,917,080	126,904,928 (99.99%)	120,665,898(95.07%)	6,239,030 (4.92%)	62,501,146 (49.25%)	62,495,526 (49.24%)
L05	145,833,598	145,811,646 (99.98%)	138,446,050 (94.93%)	7,365,596 (5.05%)	71,717,414 (49.18%)	71,685,406 (49.16%)

**Table 5:** Statistical table of biological replicate correlation.

**Sample**	**L01**	**L02**	**L03**	**L04**	**L05**
L01	1	0.879	0.7322	0.6584	0.7127
L02	0.879	1	0.8284	0.7986	0.772
L03	0.7322	0.8284	1	0.7099	0.761
L04	0.6584	0.7986	0.7099	1	0.7188
L05	0.7127	0.772	0.761	0.7188	1

### 3.2. Identification of circular RNAs (circRNAs)

**Figure 1:**
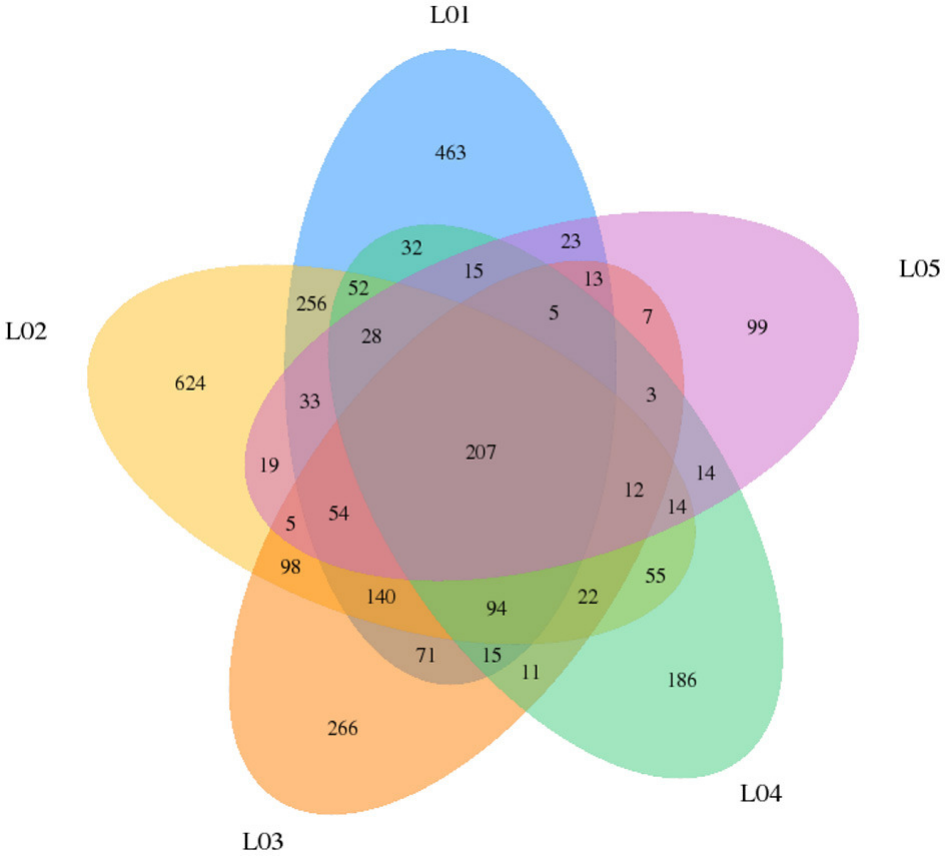
Statistical diagram of circRNA species and number expressed in each sample.

**Figure 2:**
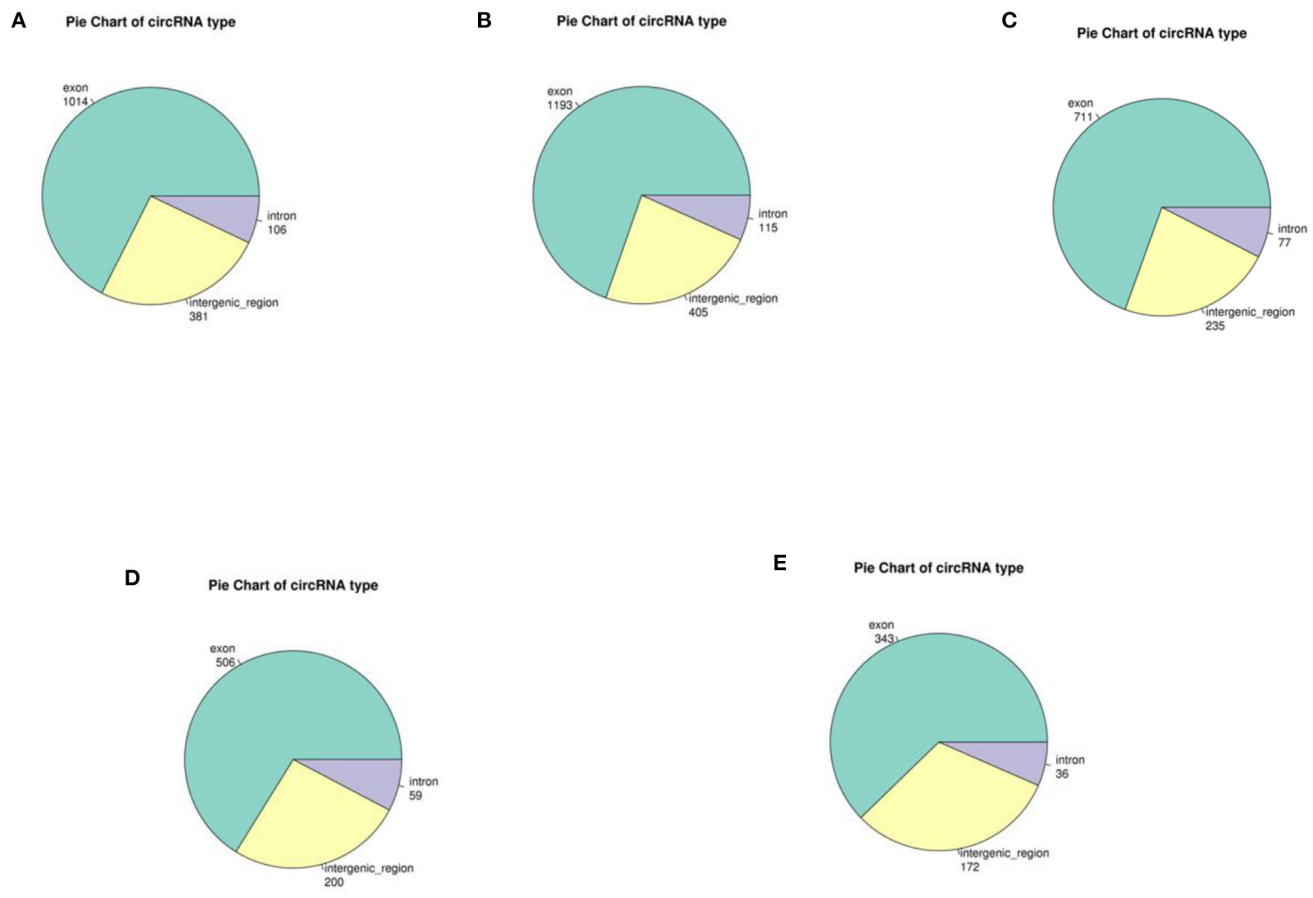
Distribution diagram of circRNA sources for each sample. Different colors represent different circRNA types, and numbers represent the proportion of CircRNA types in the total. **(A–C)** represent the source distribution of circRNA in three yak samples; **(D, E)** represent the source and distribution of circRNA in cattleyak samples.

**Figure 3:**
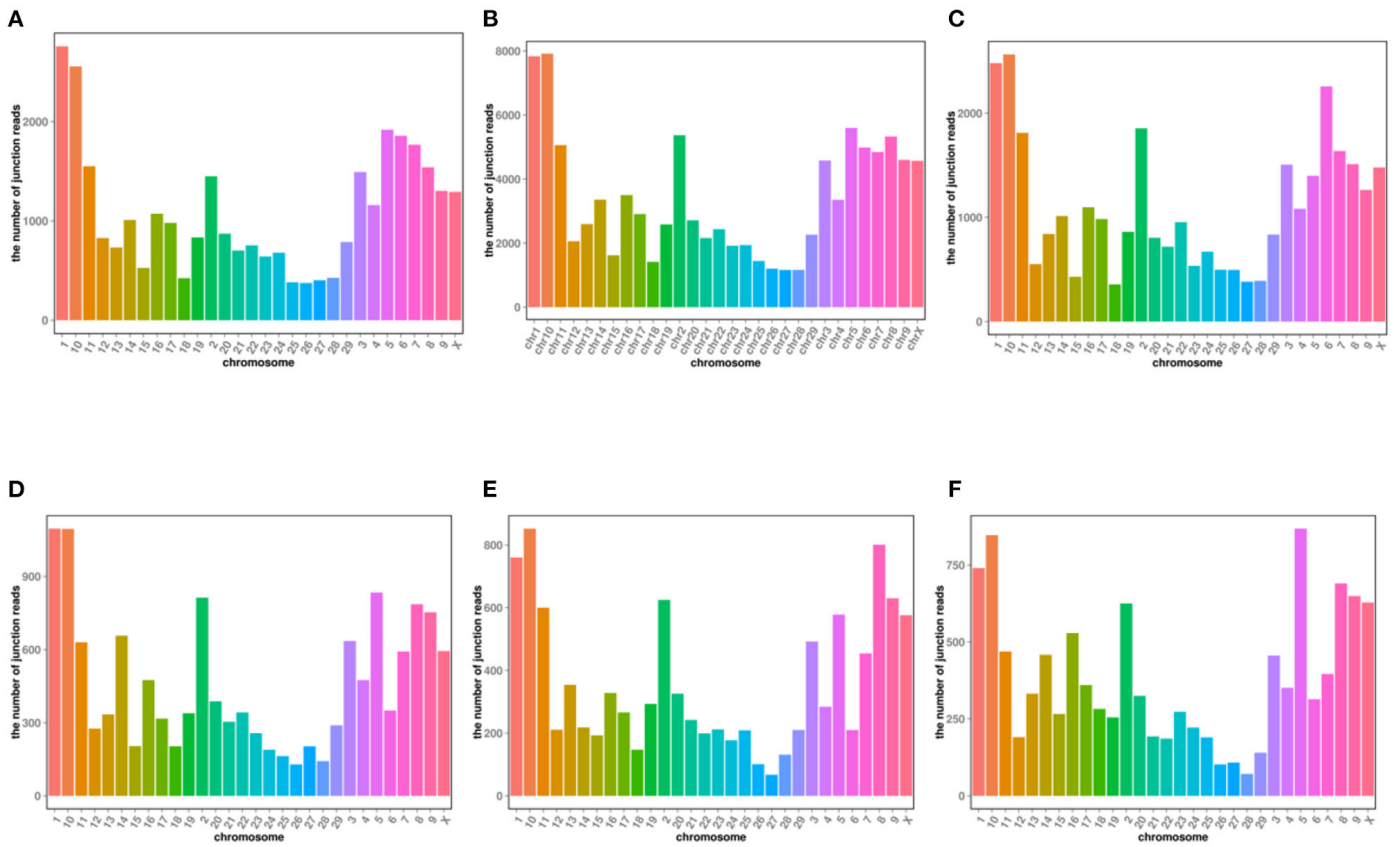
CircRNA chromosome reads distribution of each sample. **(A)** Distribution of RNA on all chromosomes; **(B–D)** distribution of circRNA on chromosomes in Yak samples; Distribution of circRNA on chromosomes in **(E, F)** cattleyak samples.

**Figure 4:**
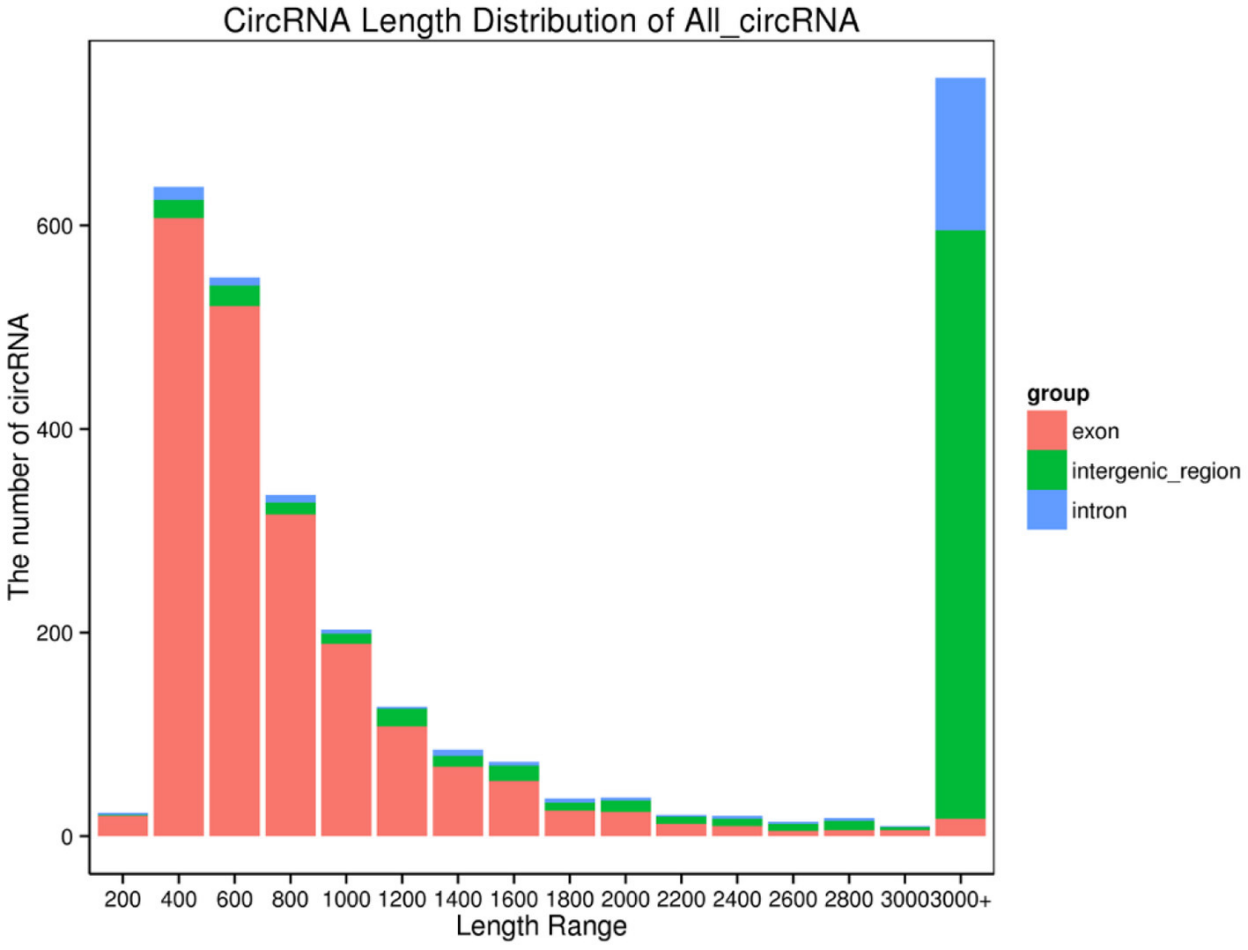
CircRNA length distribution. Abscissa, length interval; Ordinate, the number of circular RNAs in the length interval.

In yak verses cattleyak biological replicates (L01, L02, L03 vs. L04, L05), we identified a total of 1,298 candidate circRNAs by using CIRI and find-circ (8) software. 1,501, 1,713, and 1,023 circRNAs were found in three groups of yaks and 756 and 551 circRNAs were found in two groups of cattleyak (Table 6). 207 circRNAs were common among five replicates from the total identified circRNAs (Figure 1). Based on the position of circRNAs in the genome, these circRNAs were generally divided into three types: exonic, intronic and intergenic, from which most of the detected circRNAs in our study were originated from exonic regions and then intergenic regions and intronic regions respectively (Figure 2). Next, the distribution of circRNAs in different samples on different chromosomes was calculated and resulted that the mainly circRNAs were produced by chromosome 1 and chromosome 10 (Figure 3). The distribution statistics of circRNA length in each sample showed that those circRNAs who had ranged 200–2,200 bp in length were mainly originated from exons while those circRNAs ranged >2,600 were mainly originated from intergenic regions (Figure 4).

**Table 6:** Statistics of circRNA number.

**Sample**	**Total reads**	**Total circRNA**
L01	33,254	1,501
L02	33,069	1,713
L03	13,864	1,023
L04	10,746	765
L05	11,516	551

### 3.3. Differential expression profiling of circRNAs

**Figure 5:**
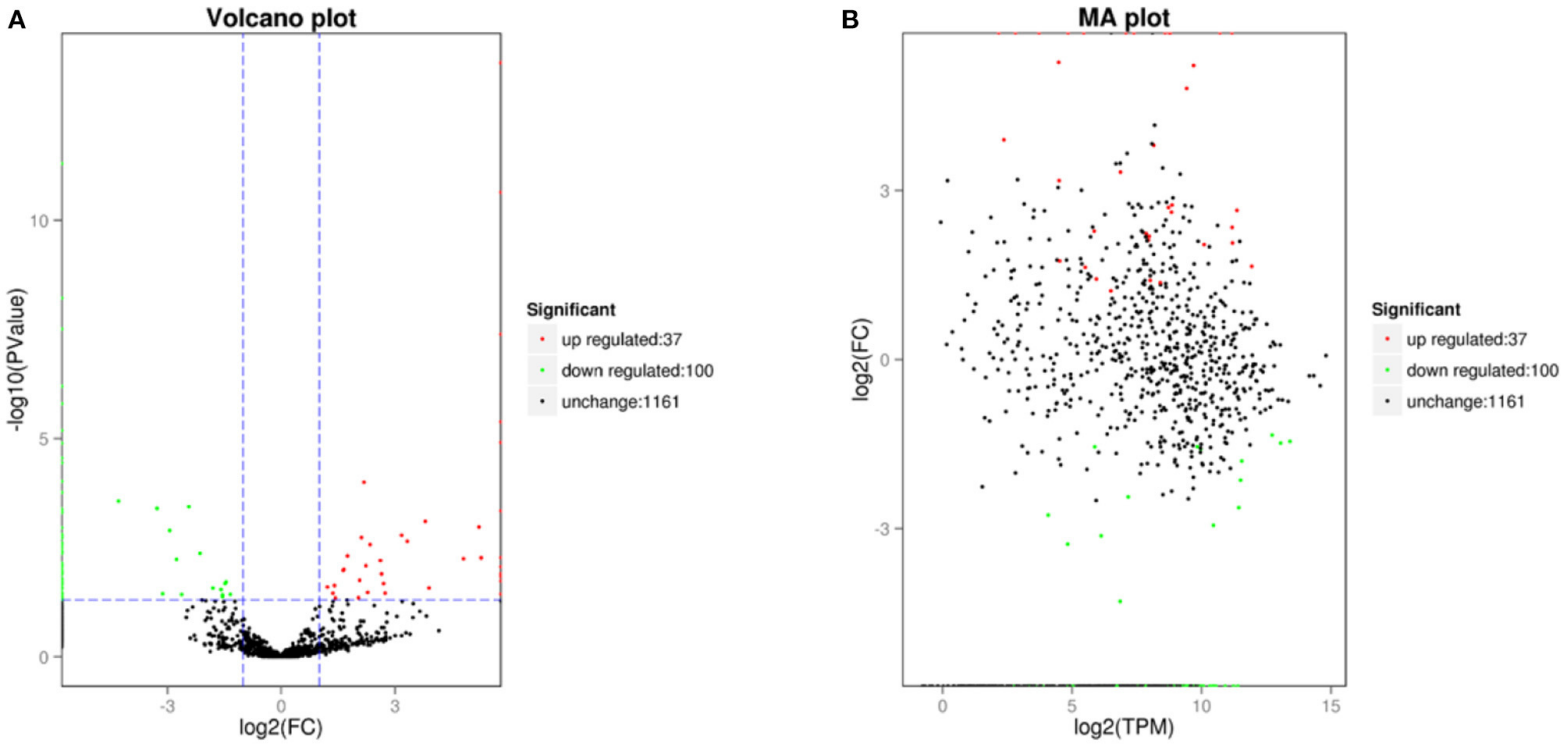
DE circRNAs plot. **(A)** is a volcano diagram of DE circRNA, that each point in the diagram represents a circRNA. **(B)** is a DE circRNA MA plot and each point in the diagram represents a gene.

**Figure 6:**
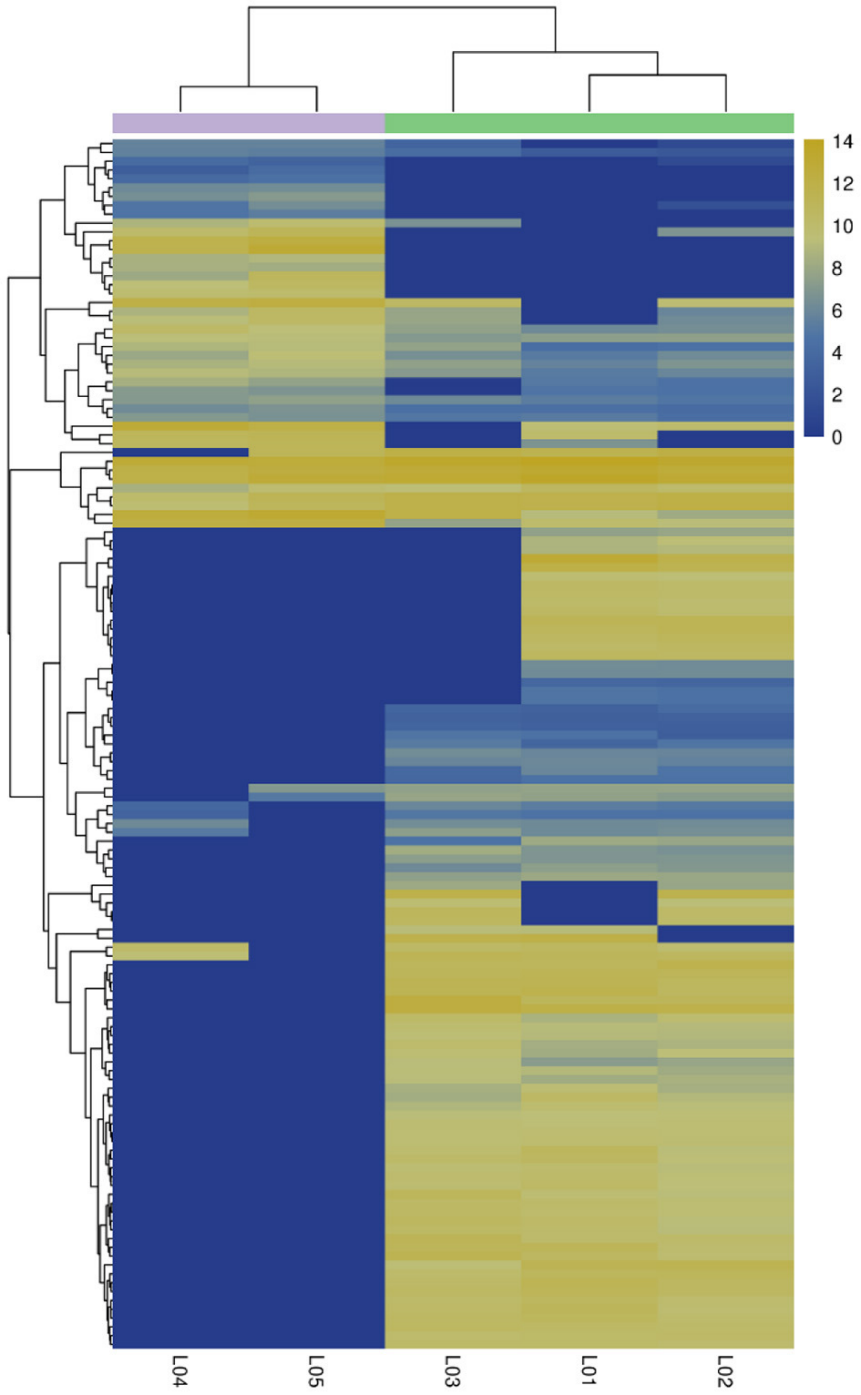
Correlation heat map. Different columns in the figure represent different samples, and different rows represent DE circRNAs. The color represents the expression level of circRNA in the sample (log2tpm + 1).

In the differential expression circRNA detection process, we had set fold difference ≥2 and *p-*value < 0.05 as the screening criteria. The fold difference described the ratio of expression between two samples (groups), and a significant *p-*value was used as a screening key indicator of DE circRNAs. A total of 137 circRNAs were DE circRNA, of which 100 were u*p-*regulated and the remaining 37 were down-regulated (see Tables 7, 8, Figure 5). Furthermore, hierarchical clustering analysis of DE circRNAs indicates that circRNA richness in yaks is higher than in cattleyak in the heat map (Figure 6).

**Table 7:** Statistics of the number of DE circRNAs.

**DEG_Set**	**All DE circRNAs**	**Up-regulated**	**Down- regulated**
L01_L02_L03 vs. L04_L05	137	37	100

**Table 8:** TOP20 with significantly differentially expressed circRNAs.

**CircRNA ID**	**log2FC**	**mRNA ID**	**miRNA**	**Regulated**	**nr_annotation**	**Genes**
9:34348787| 34358839	5.2163	ENSBTAG00000026673		Up	PREDICTED: importin subunit alpha-6 [Bos taurus]	KPNA5—karyopherin subunit alpha 5 [Bos taurus]
29:1454482| 1481574	4.8099	ENSBTAG00000035110		Up	TPA: hypothetical LOC537776 [Bos taurus]	DEUP1—deuterosome assembly protein 1 [Bos taurus]
6:90861625| 90885587	4.1576	ENSBTAG00000043960	bta-miR-149-5p	Up	Probable bifunctional methylenetetrahydrofolate dehydrogenase/cyclohydrolase 2 [Bos taurus]	MTHFD2L—methylenetetrahydrofolate dehydrogenase (NADP+ dependent) 2 like [Bos taurus]
X:132347491| 132372215	3.8284	ENSBTAG00000009420		Up	Sex comb on midleg-like protein 2 [Bos taurus]	SCML2—Scm polycomb group protein like 2 [Bos taurus]
13:81601022| 81603970	3.4827	ENSBTAG00000007917	bta-miR-370	UP	PREDICTED: teashirt homolog 2 isoform X3 [Bos taurus]	-
17:59393295| 59396057	3.3986	ENSBTAG00000007855	unconservative_18_ 753006	Up	PREDICTED: V-set and immunoglobulin domain-containing protein 10 isoform X1 [Bos taurus]	VSIG10L—V-set and immunoglobulin domain-containing protein 10
24:41588500| 41590938	3.2892	ENSBTAG00000009459	unconservative_19_ 848161	Up	PREDICTED: microtubule cross-linking factor 1 isoform X1 [Bos taurus]	MTCL1—microtubule crosslinking factor 1
5:43392396| 43445741	3.1904	ENSBTAG00000003748	unconservative_19_ 851450	Up	PREDICTED: CCR4-NOT transcription complex subunit 2 isoform X2 [Tupaia chinensis]	CNOT2—CCR4-NOT transcription complex subunit 2
3:78748144| 78779079	3.1740	ENSBTAG00000005442	bta-miR-2366	Up	TPA: WD repeat domain 78 [Bos taurus]	DNAI4—dynein axonemal intermediate chain 4
3:50357241| 50436194	3.0038	ENSBTAG00000027321	unconservative_19_ 835105	Up	PREDICTED: coiled-coil domain-containing protein 18 isoform X4 [Bos taurus]	CCDC18—coiled-coil domain containing 18
17:55129073| 55143113	−0.0058	ENSBTAG00000019278	bta-miR-2320-5p	Down	TPA: kinetochore associated 1 [Bos taurus]	KNTC1—kinetochore associated 1
20:2680598| 2714639	−0.0166	ENSBTAG00000024801		Down	PREDICTED: ran-binding protein 17 isoform X1 [Bubalus bubalis]	RANBP17—RAN binding protein 17
14:63246430| 63253538	−0.0177	ENSBTAG00000021449		Down	PREDICTED: DDB1- and CUL4-associated factor 13 [Bison bison bison]	DCAF13—DDB1 and CUL4 associated factor 13
8:23963190| 23963897	−0.0203	ENSBTAG00000014124		Down	Protein AF-9 [Bos taurus]	MLLT3—MLLT3 super elongation complex subunit
1:119954271| 119963954	−0.0412	ENSBTAG00000002395	unconservative_18_ 739965	Down	Hermansky-Pudlak syndrome 3 protein [Bos taurus]	HPS3—HPS3 biogenesis of lysosomal organelles complex 2 subunit 1
29:1011931| 1015558	−0.0437	ENSBTAG00000003552	unconservative_1_ 62488	Down	Mediator of RNA polymerase II transcription subunit 17 [Bos taurus]	MED17—mediator complex subunit 17
16:38409896| 38463364	−0.0460	ENSBTAG00000017727		Down	Kinesin-associated protein 3 [Bos taurus]	KIFAP3—kinesin associated protein 3
17:56316446| 56345720	−0.0547	ENSBTAG00000020584		Down	Intraflagellar transport protein 81 homolog [Bos taurus]	IFT81—intraflagellar transport 81
9:89715565| 89727906	−0.0704	ENSBTAG00000004099	unconservative_ GJ057352.1 _2224595	Down	PREDICTED: coiled-coil domain-containing protein 170 isoform X1 [Bos taurus]	CCDC170—coiled-coil domain containing 170
25:38549674| 38550026	−0.0722	ENSBTAG00000005908	bta-miR-149-5p		Radial spoke head 10 homolog B [Bos taurus]	RSPH10B—radial spoke head 10 homolog B (Chlamydomonas)

### 3.4. circRNA target gene prediction

circRNAs can regulate important biological processes by regulating the circRNA–miRNA–mRNA pathway. Therefore, we performed circRNA target gene prediction using RNAhybrid 2.1.2 software. Out of the total 137 DE circRNAs, 79 DE circRNAs were predicted that targeted 214 miRNAs. Since circRNA contains multiple miRNA binding sites, we used the method of miRNA target gene prediction to identify the circRNA bound to miRNA and clarify the function of this part of circRNA according to the functional annotation of miRNA target gene (Table 9).

**Table 9:** miRNA target gene information.

**miRNA**	**Target**
bta-let-7b	16:13087268|13101045
bta-let-7b	6:59467638|59530018
bta-let-7b	10:76939146|76948381
bta-let-7b	16:13087268|13094724
bta-let-7b	5:86629598|86690338
bta-miR-103	3:75369818|75410730
bta-miR-103	3:75410509|75435273
bta-miR-106a	18:46155528|46202870
bta-miR-1224	16:55142914|55154544
bta-miR-1224	21:63297835|63323570

### 3.5. GO annotation and KEGG enrichment analysis of circRNA host genes

**Figure 7:**
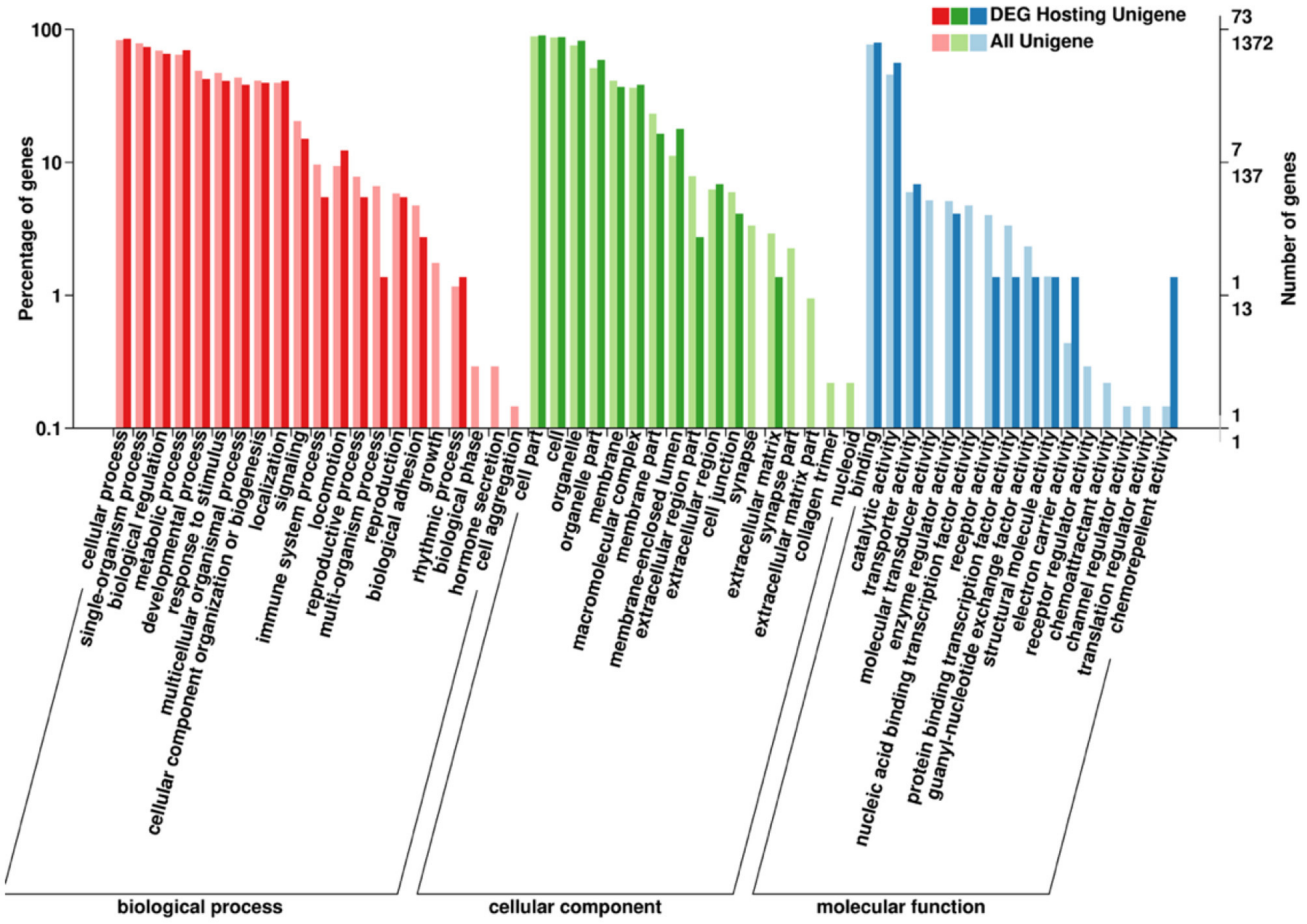
Annotated statistical diagram of circRNA-derived Gene Ontology Consortium (GO) secondary nodes. The abscissa is the go classification, the left side of the ordinate is the percentage of the number of genes, and the right side is the number of genes.

**Figure 8:**
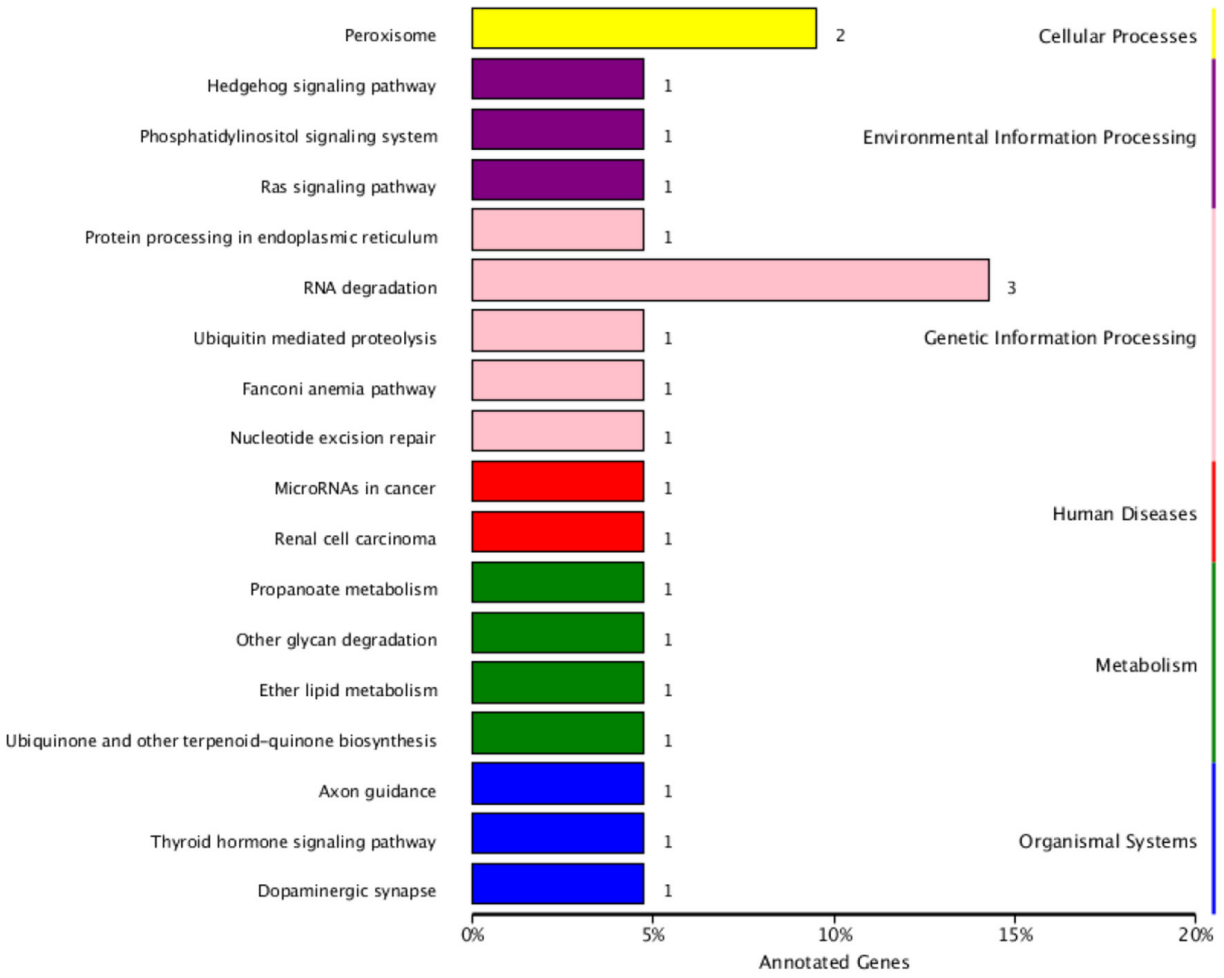
KEGG classification of circRNA-derived genes. The ordinate is the name of KEGG metabolic pathway, and the abscissa is the number of circRNA source genes annotated to this pathway and their proportion in the total number of genes annotated.

**Figure 9:**
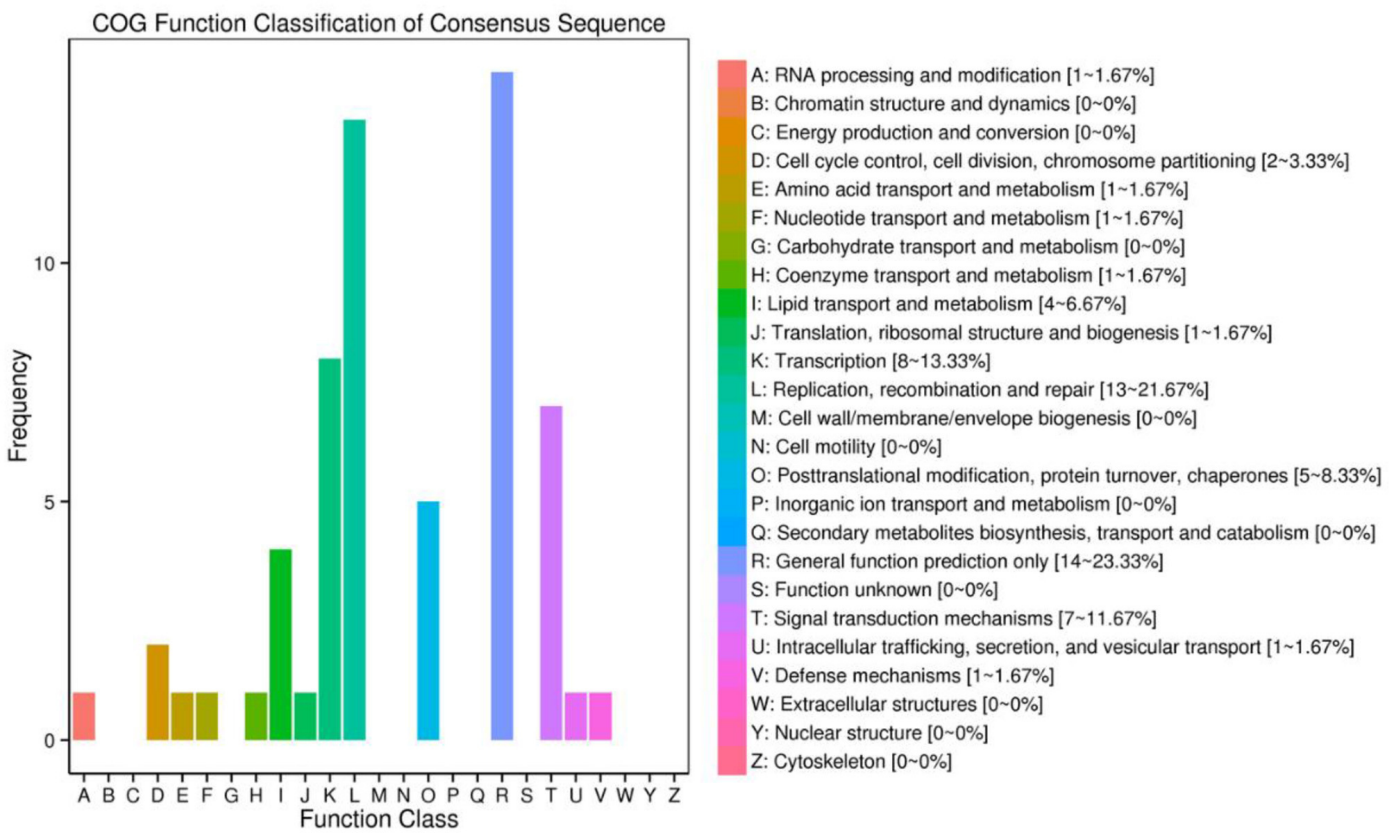
CircRNA origin gene Cluster of Orthologous Groups of proteins (COG) annotation classification statistical map. The abscissa is the classification content of COG, and the ordinate is the number of genes.

The functional annotation of the source genes of circRNA was carried out to discover new circRNA for the yak and cattleyak to complement the original genome annotation information (Table 10). We conducted GO and KEGG pathway analysis on the genes producing the DE circRNAs. The GO annotation was arranged in three main categories, namely Cellular Component (CC), Molecular Function (MF), and Biological Process (BP). According to BP category, the DE circRNA host genes were involved in metabolic process, developmental process, immune system process, reproductive process, reproduction, biological adhesion, and growth; according to CC category, the DE circRNA host genes were involved in membrane-enclosed lumen, extracellular region and organelle; according to MF category, the DE circRNA host genes were involved in chemorepellent activity, eletron carrier activity and catalytic activity (Figure 7). Different gene products interact with each other to perform biological functions and the pathway annotations of host genes of circRNAs which could provide a better understanding of the functions of these genes. KEGG enrichment analysis showed that 18 pathways were related to circRNAs, mainly including RNA degradation and peroxisome (Figure 8). COG classification analysis of circRNA-derived genes showed that the genes were mainly related to replication, recombination and repair process (Figure 9).

**Table 10:** Statistical table of source genes of annotated circRNA.

**DEG Set**	**Annotated**	**COG**	**GO**	**KEGG**	**KOG**	**Swiss-Prot**	**eggNOG**	**nr**
L01_L02_L03 vs. L04_L05	81	38	73	56	58	72	81	81

### 3.6. Validation of DE circRNAs expression by qPCR

**Figure 10:**
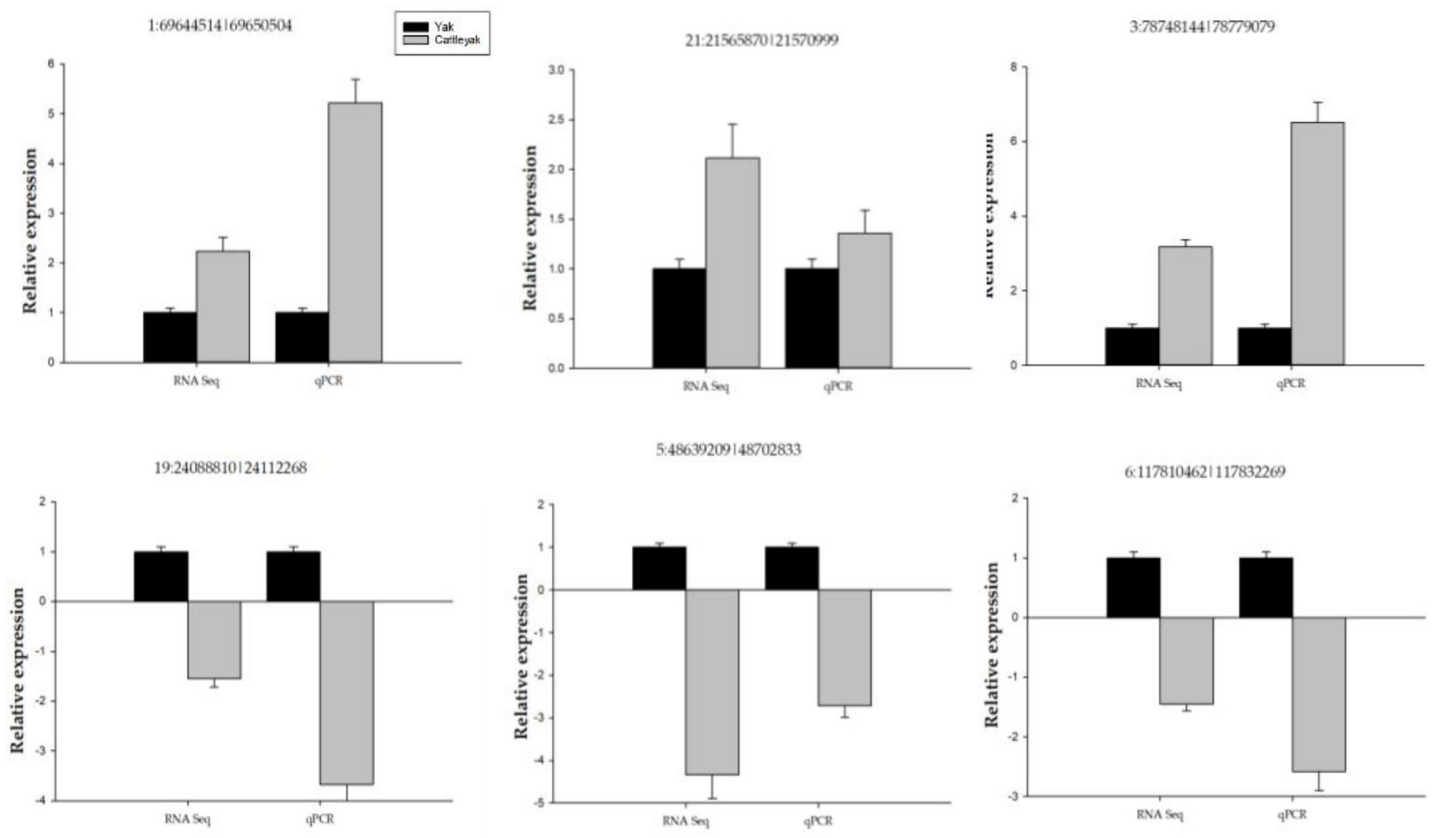
Shows qPCR validation of some selected DE circRNAs. The y-axis indicates the relative expression of six DE circRNAs between yaks and cattleyak.

We performed qPCR validation on six DE circRNAs (Table 1), and although the folding rates differed, the overall trend was consistent, proving that our sequencing data was correct (Figure 10). At the same time, combined with previous articles (29, 30) on the expression of DE miRNA in yak and cattleyak epididymal tissues, the relationship chain between circRNA-miRNA-mRNA was confirmed.

## 4. Discussion

In previous studies, scientists have conducted a variety of studies on yak and cattleyak epididymal tissues (31), utilizing proteomics (15) and high-throughput sequencing techniques (29). These studies geared toward explaining the possible mechanism of cattleyak infertility in the epididymal region. CircRNAs are one of the emerging non-coding RNAs. Over the years, their physiological functions in various tissues of mammalian species have been studied. However, since its discovery in the 1980s, its exploration in mammalian epididymis is still lacking behind. Therefore, we intend to explore its potential role in the epididymis of yak and cattleyak for the first time. Another main factor of our motivation is to uncover the molecular mechanisms underlying the process of sperm maturation and cattleyak infertility.

In this study, we identified a total of 1,298 circRNAs between the epididymis of yak and cattleyak by employing the RNA-sequencing technique. Of the total 1,298 circRNAs, 137 circRNAs were differentially expressed and among them 100 were u*p-*regulated while the remaining 37 were down-regulated in cattleyak. The circRNAs identified in the epididymal tissues of yak and cattleyak were less than that in testicular tissues, mammary gland tissues, adipose tissues and skeletal muscle tissues of other bovine animal species (23, 32–34). Furthermore, 1,753 CircRNAs were detected in the testis of newborn (1 week old) and adult (4 years old) Qinchuan yaks (23). There were significant differences in the expression of 4,248 circRNAs between neonatal and adult yak testis (23). Among these circRNAs, 2,225 were u*p-*regulated, and the relative expression of circRNAs in chromosomes 1 and 10 was observed higher indicating that it was consistent with our results (23). A total of 7,203 circRNAs were found in the study of yak adipocyte differentiation on days 0, 2 and 12, of which 4,139 circRNAs were detected on day 0, 4,755 on day 2 and 5,505 on day 12, whereas 2,737 circRNAs were detected at all three time points (35). In the analysis of circRNA expression characteristics of testicular samples from 6-month-old bulls, a total of 17,013 circRNAs were identified, of which 681 circRNAs were differentially expressed. Of these DE circRNAs, 579 were u*p-*regulated and 103 were down regulated in calf and bull testis (36). In consistent with previous reports, we identified that most of the circRNAs were derived from exonic regions, then intergenic and intronic regions, respectively.

Most of the DE circRNAs detected were from chromosomes 1 and 10. Related literatures showed that bovine chromosome 1 was related to the biological functions of α-1 crystal protein (CRYA1), γ -S crystal protein (CRYG8), superoxide dismutase I (SOD1) and uridine-monophosphates (UMPS) (24). In addition, a genetic map of DNA loci on Bovine Chromosome 1 occurred in comparison with that of other mammals during the evolutionary process (25). Chromosome 10 is associated with beef odor (25) and marbling of bovine muscle (26).

In DE circRNA genes, SCML2 (27) is a meiotic chromatin related protein encoded by the X chromosome. In mouse studies, SCML2 was found to be associated with phosphorylated H2AX. The loss of SCML2 in mice will lead to sperm production defects, resulting in a sharp decline in sperm production. SCML2 binds to the hypomethylation promoter rich in H3K4me2/3 in undifferentiated sperm to promote H3K27me3, thereby affecting sperm differentiation and the totipotency of oosperm (37). SCML2 was identified as a key regulator of heterochromatin tissue in spermatogenesis (38). RANBP17 (39) may play a role in the inactivation of sex chromosomes during the meristematic stage of spermatogenesis, as well as in the intragranular transport during spermatogenesis. PAFAH1B1 is a highly conserved protein, and studies in mice and other domesticated animals have found that *PAFAH1B1* has an important relationship with spermatogenesis and embryonic development (40). *MSRB3* plays an important role in ossification and fat deposition in cattle (41). *KALRN* is involved in synaptic regulation (42).

CircRNAs have demonstrated closer relationship with miRNAs and are known to be miRNA sponges. miRNAs are notably known as the regulators of genes at post-transcriptional level. Through circRNA-miRNA-mRNA pathway, the circRNAs regulate most important parameters of sperm maturation, such as circRNA_002581-miR-122-Slc1a5, and circRNA_007585-miR-326- UCP2 (43). CircRNAs function by binding to miRNA. Therefore, we used miRNA target gene prediction method to identify CircRNA bound to miRNA (Table 9). The results showed that bta-let-7b in the identification results were related to lipid metabolism and mitochondrial activity (44). In the study of vitrified bovine embryos, it was found that bta-let-7b was a potential target for cell pluripotency, cell programming, survival rate after cryopreservation, embryo quality, lipid metabolism and mitochondrial activity, which were related to miRNA targeting lipid metabolism and mitochondrial activity genes (44). In yak and cattleyak studies, downregulation of the bta-let-7 family, bta-miR-125, and bta-miR-23a may impair RA-induced sperm division (10). The target mRNA ENSBTAG000000022360 of circRNA 5:86629598|86690338 corresponds to the gene *SOX5* associated with Polish Holstein-Friesian Bulls' semen quality (45), so there may be a 5:86629598|86690338 to bta-let-7b to SOX5 relationship chain in this study. Bta-mir-103 may be related to heat stress (46) and sperm motility (47) after cryopreservation in cattle and the expression of bta-mir-103 in yak and cattleyak testis is also different (10). Bta-mir-106a is widely expressed in yaks (48), and is related to TGFβ1/smad signaling pathway (49). Bta-mir-1224 is a fibroblast related miRNA that can inhibit the expression of *COL11A2, FGFR3, ISLR2* and *MYH3* genes in muscle tissue of Xinjiang brown cattle (50).

According to KEGG pathways analysis results (Figure 8), most of the DE circRNA came from peroxisome-related genes (51), and antioxygenation (52) and are closely related to the peroxisomal lipid metabolism. Peroxidase is also involved in regulating signal homeostasis of spermatogenesis (53), which may also be one of the reasons leading to the difference in reproduction between yak and cattleyak. From COG annotation analysis (Figure 9), it is speculated that circRNAs may be involved in signal transduction. The literatures also indicate that circRNAs have certain effects on signaling pathways, such as inhibition of Wnt/β-catenin signaling pathway by circRNA-ITCH (54) and regulation of circRNA-000911/ Mir-449a pathway in breast cancer (55). Furthermore, vesicle transport of circRNAs *in vivo* may indicate that these RNAs may also function as signaling molecules (24).

Angiotensin converting enzyme (ACE) is a male sterility-related enzyme (56). Studies in male mice have found that *ACE1* ^−/−^ male mice are infertile (57). ACE can be synthesized endogenously in the epididymis, independent of the testis (58). The activity of ace in epididymal epithelial cells depends on androgen activity (59). In the study of renin-angiotensin-aldosterone system (RAAS), it was found that the expression of ace was affected by circRNA. The interaction between hsa_ circ_ 0122153/hsa_ mir_ 483_ 3p affects the expression of Ang II, which can promote ACE and inhibit the expression of angiotensin converting enzyme 2 (ACE2) in neurons through p38 MAPK and erk1/2 signaling pathways. In addition, has_ mir_ 483_ 3 can also act directly on ACE (60). In our study, we found that the target miRNA (bta-let-7b) of the differentially expressed circRNA was related to fat synthesis, and related studies showed that ACE2 deficiency showed lipid accumulation and mitochondrial dysfunction in skeletal muscle (61). In subcutaneous fat, high-fat diet inhibited ACE2 mRNA (62). Besides, studies on the epididymal fat pad of normal mice showed that physiologically activated NKT cells express high levels of intracellular IFN-g (63). The epididymal fat pad is a warehouse of immune cells in the epididymis, especially under inflammatory conditions (64). To sum up, these findings imply that the circRNA target genes we predicted may be related to ACE, and the ACE in the epididymis of yak and cattleyak may affect their fertility by acting on the epididymis adipose tissue.

## 5. Conclusion

In conclusion, we identified DE circRNA in epididymis of yaks and cattleyak, and analyzed the source genes of DE circRNA by bioinformatics. So far, there are few reports about circRNA in epididymis of yaks and cattleyak. This study will lay a foundation for solving the infertility of cattleyak, and breeding of yaks and cattleyak in the future.

## Data availability statement

The datasets presented in this study can be found in online repositories. The names of the repository/repositories and accession number(s) can be found below: https://www.ncbi.nlm.nih.gov/, PRJNA883995.

## Ethics statement

The animal study was reviewed and approved by Ethics Committee: Biological and Health Ethics Committee of Southwest University of Science and Technology affiliation: Southwest University of Science and Technology. Written informed consent was obtained from the owners for the participation of their animals in this study.

## Author contributions

CL: writing—original draft and conceptualization. YY: writing—review and editing. CP: data curation. MA: methodology. KS: visualization. PW: investigation. MP: supervision. KL: software. YW: methodology and supervision. WZ: funding acquisition. All authors contributed to the article and approved the submitted version.
